# Zebrafish skeleton development: High resolution micro-CT and FIB-SEM block surface serial imaging for phenotype identification

**DOI:** 10.1371/journal.pone.0177731

**Published:** 2017-12-08

**Authors:** Jeremie Silvent, Anat Akiva, Vlad Brumfeld, Natalie Reznikov, Katya Rechav, Karina Yaniv, Lia Addadi, Steve Weiner

**Affiliations:** 1 Department of Structural Biology, Weizmann Institute of Science, Rehovot, Israel; 2 Department of Chemical Research Support, Weizmann Institute of Science, Rehovot, Israel; 3 Department of Materials, Institute of Biomedical Engineering, Imperial College London, London, United Kingdom; 4 Department of Bioengineering, Institute of Biomedical Engineering, Imperial College London, London, United Kingdom; 5 Department of Biological Regulation, Weizmann Institute of Science, Rehovot, Israel; Universite de Nantes, FRANCE

## Abstract

Although bone is one of the most studied living materials, many questions about the manner in which bones form remain unresolved, including fine details of the skeletal structure during development. In this study, we monitored skeleton development of zebrafish larvae, using calcein fluorescence, high-resolution micro-CT 3D images and FIB-SEM in the block surface serial imaging mode. We compared calcein staining of the skeletons of the wild type and nacre mutants, which are transparent zebrafish, with micro-CT for the first 30 days post fertilization embryos, and identified significant differences. We quantified the bone volumes and mineral contents of bones, including otoliths, during development, and showed that such developmental differences, including otolith development, could be helpful in identifying phenotypes. In addition, high-resolution imaging revealed the presence of mineralized aggregates in the notochord, before the formation of the first bone in the axial skeleton. These structures might play a role in the storage of the mineral. Our results highlight the potential of these high-resolution 3D approaches to characterize the zebrafish skeleton, which in turn could prove invaluable information for better understanding the development and the characterization of skeletal phenotypes.

## Introduction

The zebrafish, a species belonging to the Cyprinidae family, is a well-studied vertebrate developmental model because of its basal phylogenetic location, because of the high degree of homology between human and zebrafish genes [1] and organ systems [2] and also because of the optical clarity of its embryos and larvae, allowing *in vivo* observations during development [3–5]. In addition, the ability to manipulate the embryo enables the use of different genetic techniques, such as reverse-genetic approaches that allow the functional study of a missing gene, or the use of transgenic approaches that enable the creation of zebrafish expressing fluorescent proteins [6]. Clearly the insights gained from genetic manipulations are directly related to our ability to identify and characterize the resulting phenotype.

Skeleton development is generally documented by using histological stains, such as calcein green, calcein blue, alcian blue or alizarin red [7–15]. These stains bind calcium and calcium containing mineral, albeit not exclusively [16–18]. Radiography has also been used to obtain 2D projections of only the mineralized regions [19,20]. Micro-CT has been used for monitoring adult skeletons at relatively low resolution [21], and synchrotron based micro-CT has been used for high resolution studies of the teeth (0.6 microns)[22,23]. Here we show that a laboratory based micro-CT can provide 3D high resolution images, as well as volume and mineral density quantification. The micro-CT data are subtly different from the calcein fluorescence data, and their comparison provides invaluable information for assessing skeletal phenotypes in larval zebrafish. To go further in the characterization of the skeleton, the serial focused ion beam/scanning electron microscopy (FIB-SEM) was also used to 3D visualize and quantify the bone, as the lacuno-canalicular network [24], the tendon-bone insertion [25] or the collagen network [26–30]. with a resolution in the nanometric scale.

We report several previously unidentified aspects of the zebrafish skeleton. Moreover, we illustrate the effectivity of this approach, by comparing wild type to albino mutants, as the latter are widely used in developmental studies on the assumption that in terms of skeletal development, they are similar, if not identical to the wild type.

We first document wild type skeletal development using, first, a widely used fluorescence dye; calcein, and compare these images to high-resolution micro-CT 3D images to validate the use of high resolution (around 0.6 micron) micro-CT for characterizing larval skeletal phenotypes. Calcein is known to chelate calcium ions both in solution and in the mineral bulk [10]. Therefore, calcein fluorescence does not only detect mineral in the bones as is often assumed [10], but also calcium ion concentrations at other locations. For example, the intestinal tract also fluoresces strongly [10], presumably because the intestinal tract contains high concentrations of calcium due to the food. Micro-CT faithfully maps the distributions of the dense mineral phases of the bones, the teeth and the calcium carbonate mineral of the otoliths, and therefore monitors mineralization per se in the development of the skeleton. To characterize further aspects of the development of the bones, we also used the dual beam FIB-SEM in the “slice and view” (or serial surface view SSV) mode, as previously described [26]. This high-resolution approach allowed us to observe new features in the tail and the notochord.

## Materials and methods

### Breeding and collecting of zebrafish

Zebrafish were bred and maintained in a controlled environment at 28°C as previously described [4]. The *nacre* mutant is characterized by the absence of melanocytes due to a mutation in the *mifta* gene [31]. All the experiments were carried out according to the guidelines and approved by the Weizmann Institute Animal Care and Use Committee. Embryos were obtained by placing 5–6 females and 5–6 males in a spawning tank. Eggs were collected and embryos were raised in water at 28 ± 0.5°C in an incubator for 6 days, at which time they were transferred to a normal water tank and maintained until analysis.

### Calcein staining

The larvae were immersed in a 0.2% calcein solution (Sigma-Aldrich) (pH 6.8) for 25 min and then washed three times with blue water. For *in vivo* observations, animals were anesthetized with 0.12% tricaine-metanesulfonate (MS222) in blue water. After mounting in methyl cellulose 5% (1.5%) plate, the larvae were observed using an epifluorescence stereomicroscope (Leica M167FC). Pictures were taken using Leica Application Suite imaging software version 3.7 (Leica, Wetzlar, Germany).

Confocal imaging was performed on a Zeiss LSM 780 upright confocal microscope (Carl Zeiss, Jena, Germany) with a W-Plan Apochromat × 20 objective, NA 1.0. The calcein staining was excited at 488 nm and the emission was collected at 492/577 nm. Z-stacks were acquired at 1.5 μm increments, every 1 min. Pictures were processed off-line using ImageJ (NIH) and Avizo (FEI).

4 zebrafish larvae were observed for each condition and time.

### Micro-CT scan

We imaged 3 freshly sacrificed zebrafish using a non-destructive volume visualization for each condition and at 17 dpf and 30 dpf. This method enables the visualization of soft tissues without need for chemical fixation or staining [32]. The fish were euthanized with MS222. We coated the surface of a plastic sheet 3x1x0.2 cm in size with a drop of polylysine (Sigma-Aldrich) to allow the interaction between the polyanionic surfaces of the fish and the polycationic layer of adsorbed polylysine. The sample was placed in a custom-made sample holder that allowed maintenance of high humidity around the sample and was observed using an Xradia Micro-CT-400 (Zeiss X-Ray Microscopy, Pleasanton, CA, USA), with an X-ray source of 30 kV, current 150 μA and magnification 10X. The pixel size for the 17 dpf specimen and for the tail was 0.6x0.6x0.6 microns and for the 30 dpf specimen was 1.3x1.3x1.3 microns. 1,200 projection images were recorded with 20 sec exposure time. In order to compare all the intensities of all the acquisitions, a scale was designed using a standard phantom. In addition, a hydroxyapatite CT phantom (QRM, Möhrendorf, Germany) was used as a calibration standard for the quantification of the density of zebrafish bones. Obtained data are given as mean ± standard deviation.

### FIB-SEM block surface serial imaging

We used the dual beam FIB-SEM (FEI) in the block surface serial imaging mode to study the mineralized and demineralized fin structures. Three samples were demineralized in a solution of 2% PFA, 3% EDTA and cacodylate buffer at 0.1 M overnight on a rotating table. The preserved and demineralized samples were high-pressure frozen (HPM10; Bal-Tec) in dextran (10%), then freeze-substituted (AFS2 Leica Microsystems, Vienna, Austria) in 2% glutaraldehyde in absolute ethanol. Samples were stored at -90°C for 42h, before the temperature was slowly increased to -30°C for 24h (-2°C/h) to finally reach 0°C in 20 min (60°C/h). The samples were stained based on the OTOTO protocol [26], using osmium at 1% in ethanol for 30 min and thiocarbohydrazide at 0.5% in ethanol for 15 min both at room temperature. The samples were then embedded in Epon. After polymerization, the sample surfaces were exposed using an Ultracut Reichert microtome (Leica Microsystems, Vienna, Austria) with a diamond knife (DiATOME AG, Biel, Switzerland). SSV views were made using the Helios Nanolab 600 dual beam microscope (FEI, The Netherlands) on samples sputter-coated with gold. The focused ion beam (FIB) removes slices in the XY-plane whereas the SEM, using a mixed secondary electrons/backscattered electrons (SE/BSE) detector, scans the exposed surface from the side. A sequence of serial images is recorded to form a z-stack in the direction perpendicular to the bone cross-section. The observations on the mineralized samples were carried out with a slice thickness of 26 nm covering 12 μm in depth, over an area of 26 x 22 μm. Isometric voxel sizes were maintained during all experiments. A sequence of serial images was recorded to form a z-stack.

### Cryo-SEM

Three tails were dissected from freshly sacrificed zebrafish and immediately immersed in 10% dextran (Fluka). Before being high-pressure frozen by a HPM10 (Bal-Tec), the samples were wedged between two metal discs (3 mm diameter, 0.05 mm cavities with a flat cover above). The frozen samples were then placed on a holder in liquid nitrogen environment for freeze fracture (BAF 60; Bal-Tec). The samples were longitudinally fractured at -120°C, using a vacuum better than 5 x 10^−7^ mbar. Fractured samples were observed using an Ultra 55 SEM (Zeiss, Germany) with a secondary electron in-lens detector and a backscattered electron in-lens detector operating at 1kV, with a 10 μm aperture size of and a 1.8 mm working distance. The observations were made in the frozen-hydrated state at -120°C.

### Image analysis

Images obtained with the CT-scan and the FIB SEM were analyzed using ImageJ (NIH, USA) and Avizo 8 (FEI Vizualization Sciences Group) softwares. To analyze the bone development in the zebrafish observed by micro-CT-scan, we used various commands in Avizo. For the Label Field command, we selected for each sample the bones in each slice and aligned them one upon the other obtaining a 3-dimensional stack. Then the Label Analysis command calculated the volume for each selected sample, and the Histogram command provided the intensities for each bone. After data calibration with calibration phantoms for hydroxyapatite and aragonite, we converted intensities of every voxel in the images to mineral densities.

To analyze the FIB SEM images, we first removed the effect of charging using the FFT signal of each image belonging to the stacks. Then the contrast levels of all the images were adjusted using the plugin Enhance Local Contrast [33]. The stacks were then manually aligned using the Align Slices command in Avizo. All the dense materials were surface-rendered using the Surface View command.

The directionality analyses were searched to characterize the collagen structure of the fin and determine two parameters: the direction and the dispersion [26,27]. Briefly, the first parameter provides the azimuthal direction angle of the majority of the collagen fibrils, whereas the second parameter shows the angular dispersion, *i*.*e*. the standard deviation of this first parameter. The analysis was obtained as previously described [26], with sub-stacks of 30 images and the method of Local Gradient Orientation in the Directionality plugin [34]. All the images presented in this article in side views are in the same orientation, namely with the tail to the left and the head to the right.

### Statistical analysis

For each analysis and condition, at least 3 zebrafish larvae were used. Data are given as mean ± standard deviation.

## Results

### Development of the skeleton based on high resolution micro-CT

**Fig 1:**
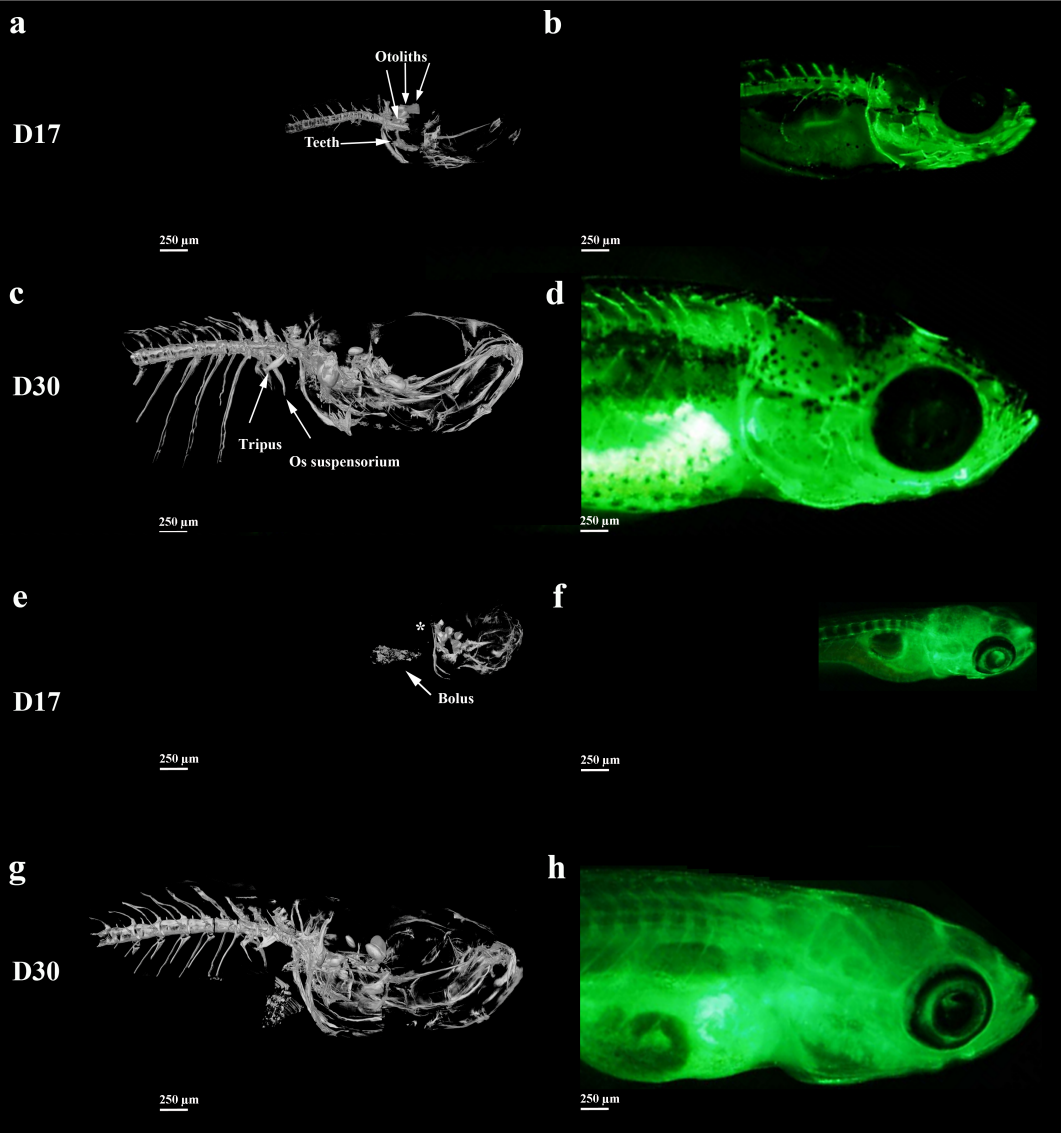
Micro-CT-scan and 3D-reconstructions (a,c,e,g) and fluorescence microscopy after calcein staining imaging (b,d,f,h) of side views of calcified skeletal structures in wild type (a,b,c,d) and *nacre* (e,f,g,h) zebrafish larvae at 17 dpf (a, b, e, f) and 30 dpf (c,d,g,h). No vertebra is observed using the CT scan observation at D17 (asterisk).

For direct comparison we examined the same calcein stained specimens in the micro-CT that were imaged using the fluorescence microscope. We focused the comparison only on the head and the first precaudal vertebrae at 17 dpf when the head and axial skeleton are fully developed based on calcein imaging, and compared these results to 30 dpf (Fig 1). At 17 dpf the micro-CT-scan of wild type larvae shows some elements of the cranium, including the cleithrum, the basioccipital process, the exoccipital, the ceratobranchial 5 including the teeth and the 3 pairs of otoliths (To have more information on the zebrafish anatomy, see S1 Fig). The first 8 vertebrae centra with their neural spines and the first ribs on the fifth vertebra are visible, as well as some elements from the Weberian apparatus. The fluorescence signal shows the presence of the same bones, but not the otoliths and the teeth. At 30 dpf, we observe all the bones in the cranium, the vertebrae with their neural spines and the first 5 ribs. At 30 dpf the Weberian apparatus is completely formed with, for example, the tripus and the os suspensorium, the otoliths are clearly seen, as are the teeth (Fig 1, S1 Video). With the fluorescence microscope, the complete axial skeleton at 30 dpf is observed, but the elements in the cranium are difficult to see. For example, the tripus and the os suspensorium that are clearly detected by micro-CT, are not visible by fluorescence (To have more information on the development of the skeleton, see S2 Fig).

**Fig 2:**
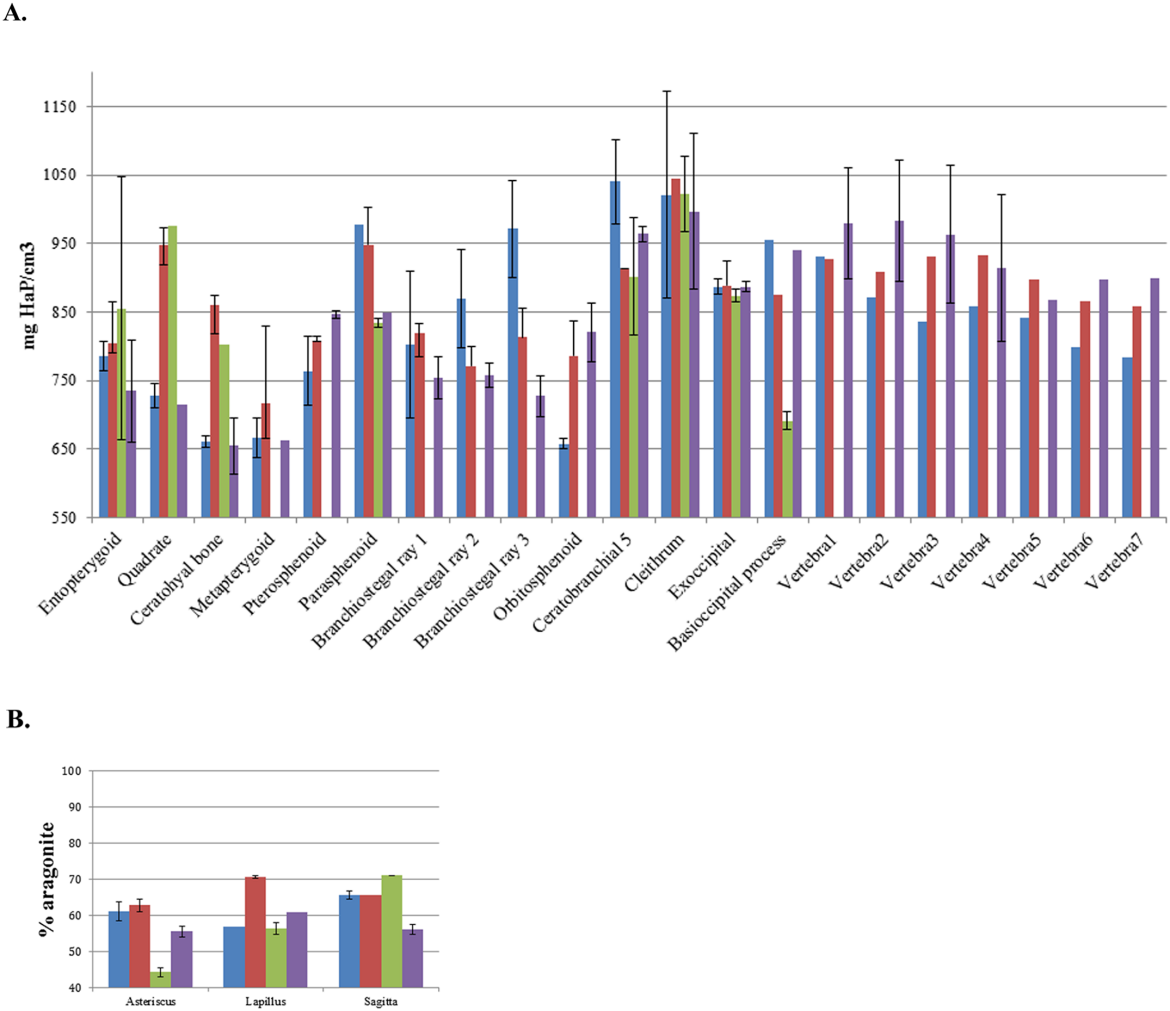
Quantification by micro-CT of elements found in the wild type and *nacre* zebrafish. **A**. Absolute mineral density quantification of some elements found in the cranium and the first 7 vertebrae at 17 dpf (blue and green) and 30 dpf (red and purple). All the bones are expressed in quantity of hydroxyapatite per cm^3^. **B**. Relative mineral density quantification by CT of otoliths at 17 dpf (blue and green) and 30 dpf (red and purple). All the bones are expressed in percentage of aragonite compared to a pure standard. Error bars = standard deviation.

The micro-CT can provide quantitative measurements of bone volume and mineral density. Such quantitative measurements could prove helpful for phenotype identification. Table 1 shows the increase in bone volume from 17 dpf to 30 dpf in the wild type. In this period of time, wild type bones increase in volume from 3 to 10 times. The 3 otolith types increase in volume from 3 to 7 times. The comparison of the mineral density values for cranial bones and vertebrae from the 17 and 30 dpf larvae respectively (Fig 2A) and for the otoliths (Fig 2B) shows that the trends during development are not always the same, due to the complex relations between rate of volume increase and rate of mineralization within the forming bone. Indeed, we observed for example that the basioccipital process at 17 dpf was more mineralized in the wild type than at 30 dpf.

**Table 1:** Volume of the bones determined by CT in the wild type at 17 dpf and 30 dpf in μm^3^.

	17 dpf	30 dpf		17 dpf	30 dpf
**Asteriscus**	80,000 ± 37,000	560,000 ±6,000	**Orbitosphenoid**	14,000 ± 5,000	30,000 ± 3,000
**Lapillus**	270,000 ± 3,000	830,000 ± 45,000	**Ceratobranchial 5**	280,000 ± 56,000	2,680,000 ± 1,000
**Sagitta**	340,000 ± 36,000	1,700,000 ± 810,000	**Cleithrum**	220,000 ± 114,000	1,250,000 ± 4,000
**Entopterygoid**	140,000 ± 25,000	470,000 ± 8,000	**Exoccipital**	90,000± 5,000	690,000 ± 134,000
**Quadrate**	60,000 ± 7,000	460,000 ± 45,000	**Basioccipital process**	280,000± 1,000	1,070,000± 4,000
**Ceratohyal bone**	20,000 ± 5,000	250,000 ± 1,000	**Vertebra1**	30,000 ± 1,000	190,000 ± 4,000
**Metapterygoid**	20,000 ± 5,000	70,000 ± 9,000	**Vertebra2**	60,000 ± 1,000	160,000 ± 2,000
**Pterosphenoid**	95,000 ± 18,000	650,000 ± 117,000	**Vertebra3**	70,000 ± 5,000	330,000 ± 1,000
**Parasphenoid**	160,000 ± 3,000	490,000 ± 2,000	**Vertebra4**	70,000 ± 1,000	210,000 ± 6,000
**Branchiostegal ray 1**	10,000 ± 4,000	110,000 ± 21,000	**Vertebra5**	60,000 ± 5,000	200,000 ± 4,000
**Branchiostegal ray 2**	25,000 ± 1,000	100,000 ± 11,000	**Vertebra6**	60,000 ± 2,000	220,000 ± 2,000
**Branchiostegal ray 3**	41,000 ±2,000	110,000 ± 23,000	**Vertebra7**	50,000 ± 4,000	210,000 ± 4,000

The vertebrae in the wild type at 30 dpf are always more mineralized than at 17 dpf, except for the first vertebra. Remarkably, at 17 dpf, vertebra 1 is the most mineralized (930 mg HaP/cm^3^), compared to vertebra 2 (870 mg HaP/cm^3^); vertebrae 4, 5 and 3 have similar densities (≈ 850 mg HaP/cm^3^) and the other vertebrae have lower densities (≤ 800 mg HaP/cm^3^). These results show that, even if the mineralization of the axial skeleton begins with the formation of vertebrae 3 and 4 [35], the first two centra are more mineralized. At 30 dpf, vertebrae 1–4 all have the same mineral density, namely more than 910 mg HaP/cm^3^. The comparison of the relative mineral density for the otoliths shows that there is no difference from 17 dpf to 30 dpf for the sagitta or the asteriscus, whereas the lapillus is more dense at 30 dpf.

In the studies using transparent lines, even if the question was previously asked to know if the lack of pigments affects the biological processes [36], the transparency was not considered to disturb the wildtype phenotype of the zebrafish.

We used our complementary micro-CT–fluorescence approach to also study the *nacre* zebrafish, as it is widely used for diverse studies because of its complete lack of melanocyte. Our aim was to determine if the mutation that caused the transparency may also have affected skeletal development [31]. At 17 dpf no vertebrae are observed in the axial skeleton of *nacre* fish using micro-CT (Fig 1). This is in sharp contrast to the vertebrae that are clearly seen with calcein fluorescence. Presumably the calcein highlights free or bound ionic calcium, but not mineral. The same “extra” fluorescence is observed in the ribs, where based on the micro-CT no mineral could be identified. At 30 dpf, no differences between wild type and *nacre* were observed using the micro-CT (S4 Video). The fluorescence images clearly show the vertebrae and the ribs, but the highly fluorescing outer bones of the cranium prevent imaging of the internal bones. In conclusion this direct comparison shows major differences between the skeletal components that are mineralized as revealed by the micro-CT and the calcein fluorescence labelling of the same specimens. The latter in some cases shows fluorescence where mature mineralized extracellular matrix is absent.

When the volumes of skull bones are quantified at 17 and 30 dpf (Table 2), the increase in volume of the *nacre* is much larger than in the wild type, namely the volume increases from 3 to 58 times for the bones already formed. In addition, two of the 3 *nacre* otolith pairs increase in volume around 6 times, whereas the asteriscus increases more than 586 times. This difference is ascribed to the fact that the formation of the asteriscus is late in development (11–12 dpf) [37] relative to the other otoliths (19–22 hpf) [38]. By 30 dpf both wild type and *nacre* fish are similar in size and skeletal development based on fluorescence. The mineral densities (Fig 2c) are also similar at 30 dpf within experimental error, with 4 interesting exceptions, namely the ceratohyal bone, the quadrate, the parasphenoid and the pterosphenoid. In the first three cases, the mineral density of the *nacre* is much lower than the wild type, whereas the last one is higher. Significantly, all these bones are found in the same proximal part of the cranium.

**Table 2:** Volume of the bones determined by micro-CT in 17 and 30 dpf *nacre* fish (μm^3^).

	17 dpf	30 dpf		17 dpf	30 dpf
**Asteriscus**	700 ± 200	390,000 ± 42,000	**Orbitosphenoid**	Absent	20,000 ±4,000
**Lapillus**	120,000 ± 60,000	780,000 ± 1,000	**Ceratobranchial 5**	70,000 ± 22,000	1,070,000± 5,000
**Sagitta**	150,000 ± 72,000	970,000 ± 49,000	**Cleithrum**	60,000 ± 5,000	1,090,000 ± 1,000
**Entopterygoid**	42,000 ± 10,000	430,000 ± 172,000	**Exoccipital**	20,000 ± 1,000	600,000 ± 60,000
**Quadrate**	20,000 ± 2,000	150,000 ± 4,000	**Basioccipital process**	30,000 ± 1,000	1,820,000± 2,000
**Ceratohyal bone**	20,000 ± 6,000	90,000 ± 28,000	**Vertebra1**	Absent	300,000 ± 1,000
**Metapterygoid**	Absent	80,000 ± 3,000	**Vertebra2**	Absent	230,000 ± 1,000
**Pterosphenoid**	Absent	120,000 ± 63,000	**Vertebra3**	Absent	260,000 ± 2,000
**Parasphenoid**	60,000 ± 1,000	1,130,000 ± 3,000	**Vertebra4**	Absent	230,000 ± 3,000
**Branchiostegal ray 1**	Absent	90,000 ± 9,000	**Vertebra5**	Absent	220,000 ± 6,000
**Branchiostegal ray 2**	Absent	140,000 ± 45,000	**Vertebra6**	Absent	220,000 ± 4,000
**Branchiostegal ray 3**	Absent	200,000 ±2,000	**Vertebra7**	Absent	380,000 ± 4,000

Comparison of the relative mineral density for the otoliths shows that the otoliths of nacre zebrafish are less mineralized or as mineralized as the wild type, except for the sagitta at 17 dpf (Fig 2d).

**Fig 3:**
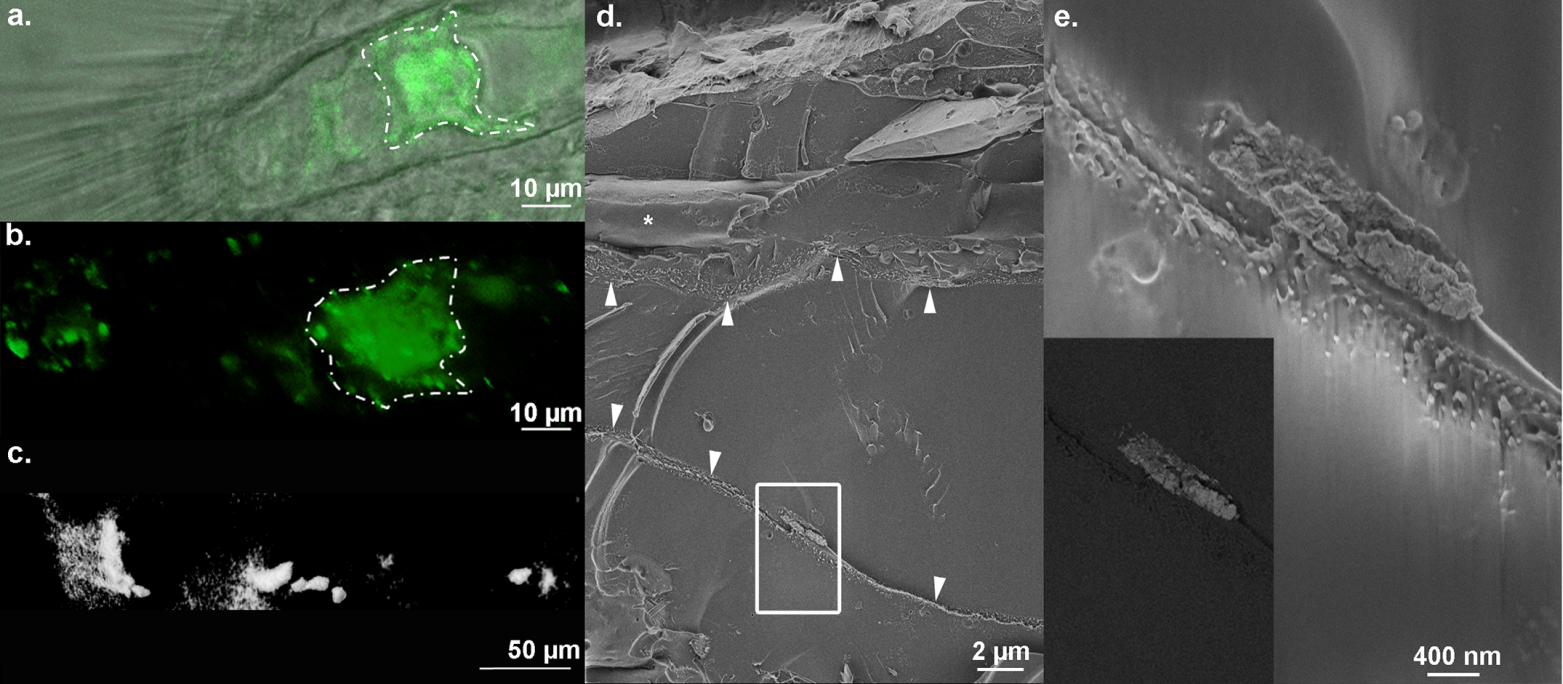
Tail area showing the unknown deposit revealed by calcein staining using confocal microscopy, micro-CT and cryo-SEM, observed in *nacre* zebrafish at 14 dpf. (**a,b**) Distal region of the body observed in top view using confocal microscopy reveals a positive staining localized in the nucleus pulposus cells (white dotted line). (**c**) In lateral view using micro-CT, we show that this positive staining is due to dense material. **d,e**. InLens secondary electron images using cryo-SEM in a longitudinal fracture of the notochord showing aggregates close to the notochord sheets (arrowheads). The collagen is found in close vicinity (white asterisks). **e**. Area magnified is delimited by the rectangle in panel **d**. Inset: Back-scattering electron imaging (BSE) of the same area observed in **e**.

Fluorescence microscopy reveals a previously unknown calcium-rich deposit in *nacre* mutants (Fig 3a and 3b). This fluorescence was observed in the distal part of the body in the same area as the large vacuolated notochord cells (the nucleus pulposus) (Fig 3b), and is present prior to the formation of the first axial skeletal element. Using confocal microscopy, we observed that this fluorescence was localized inside the cells of the 14 dpf zebrafish. Micro-CT shows that these fluorescent structures have densities similar to those of bone mineral. These structures were also observed in cryo-SEM (Fig 3d and 3e) under conditions that minimize the introduction of artifacts. In cryo-SEM they appear as large aggregates (±2 μm) close to the notochord sheet, which have a positive backscattered electron (BSE) signal, implying that they are composed of dense material.

### Newly identified mineralized features in the tail fin bone

**Fig 4:**
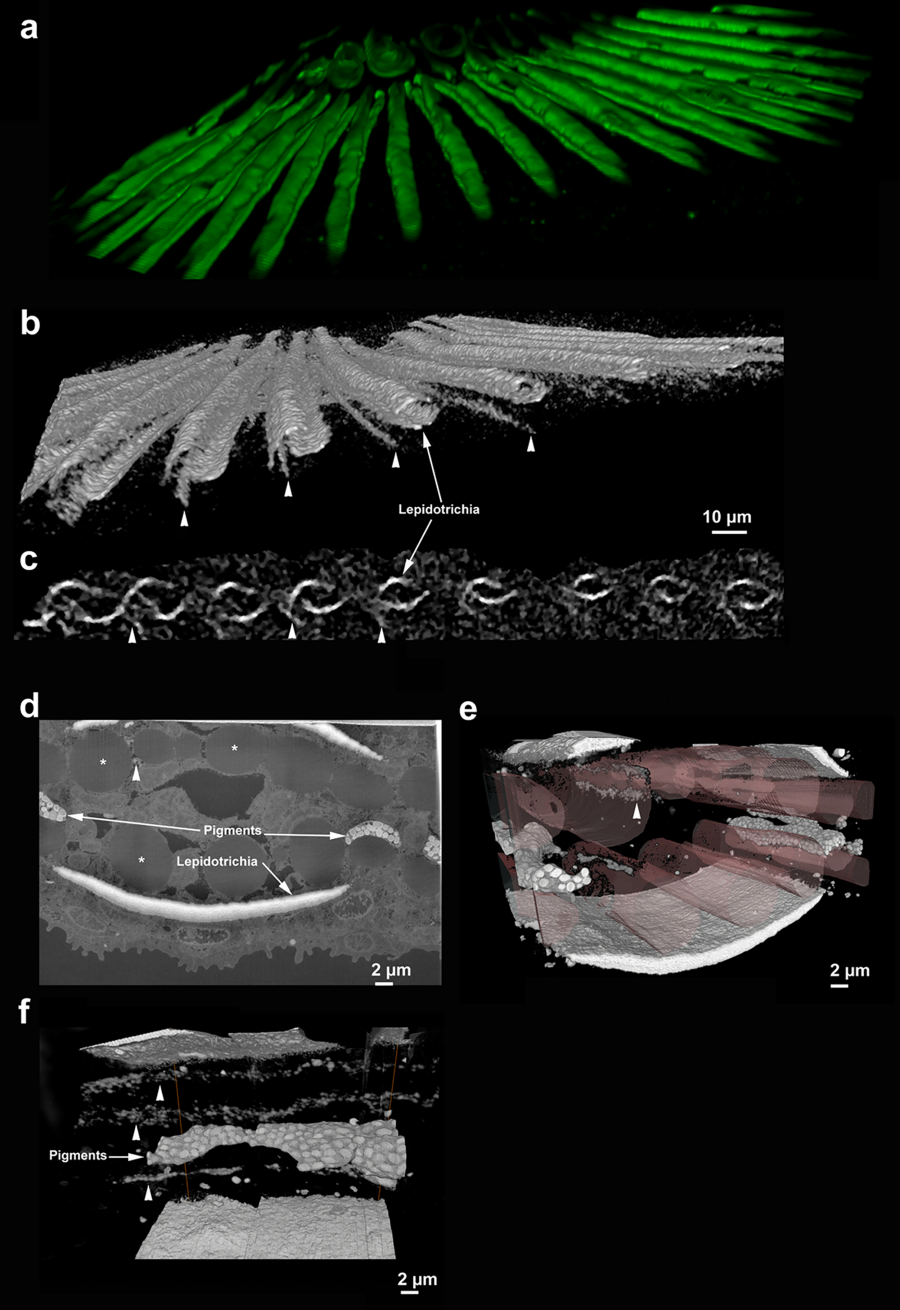
New features in the tail fin bone observed by microCT-scan and FIB-SEM. **a**. Confocal fluorescence microscopy observations of the tail stained by a calcein solution. **b)** volume rendering and **c)** cross-section of the volume rendering using CT-scan of the tail fin at 30 dpf, at a resolution of 2.5 μm, shows unknown high density structures (arrowheads). **d**, **e and f** are FIB-SEM scan and 3D reconstructions, respectively, of the forming region of a mineralized tail in cross section (**d** and **e**) and reconstructed longitudinal section (**f**) at a resolution of 20 nm. The unidentified high density structures (arrowheads) are close to the lepidotrichia (bone) and between two actinotrichia (collagen bundles) (white asterisks in d).

The micro-CT revealed unknown highly dense structures between the tail fin bones that cannot be observed by conventional *in vivo* confocal imaging (Fig 4a and 4b). These elongated dense objects are located close to the lepidotrichia and are aligned with the ventral fin bone hemi-cylinder. We used FIB-SEM in the block surface serial mode to determine the related cellular content in 3D at 10nm resolution. These features cannot be seen in micro-CT (Fig 4d, 4e and 4f) (S2 Video). Previously encountered problems of electron microscopy imaging of an untreated non-demineralized sample by FIB SEM are the absence of contrast in the biological tissue [26,27] and the possible dissolution of the mineral during processing for specimen embedding. We used an approach involving fixation, high-pressure freezing, freeze-substitution, heavy metal staining, and polymerization [39], which preserved the mineral while supporting staining. The mineralized tail including the upper and lower lepidotrichia, *i*.*e*. the dermal bony hemi-segments, appear white due to the high-density carbonated hydroxyapatite mineral deposits. The actinotrichia, *i*.*e*. the large collagenous fibers/bundles, were also observed between the hemi-cylinders. The actinotrichia appear grey. Pigment layers are observed close to the bones. The pigment layers are bright in the BSE mode due to the presence of zinc and the metal staining [40]. We observed unknown elongated structures in the micro-CT. They are located close to the lepidotrichia and between two actinotrichia, with a granular appearance. Here too they have contrast intensities similar to the bones. The localization of this unidentified structure is not the same between the Ct-scan and the FIB-SEM, but we hypothesized that this can be due to a difference of the location of the region of interest in the tail. But this unidentified structure is always in the vicinity of the bones.

**Fig 5:**
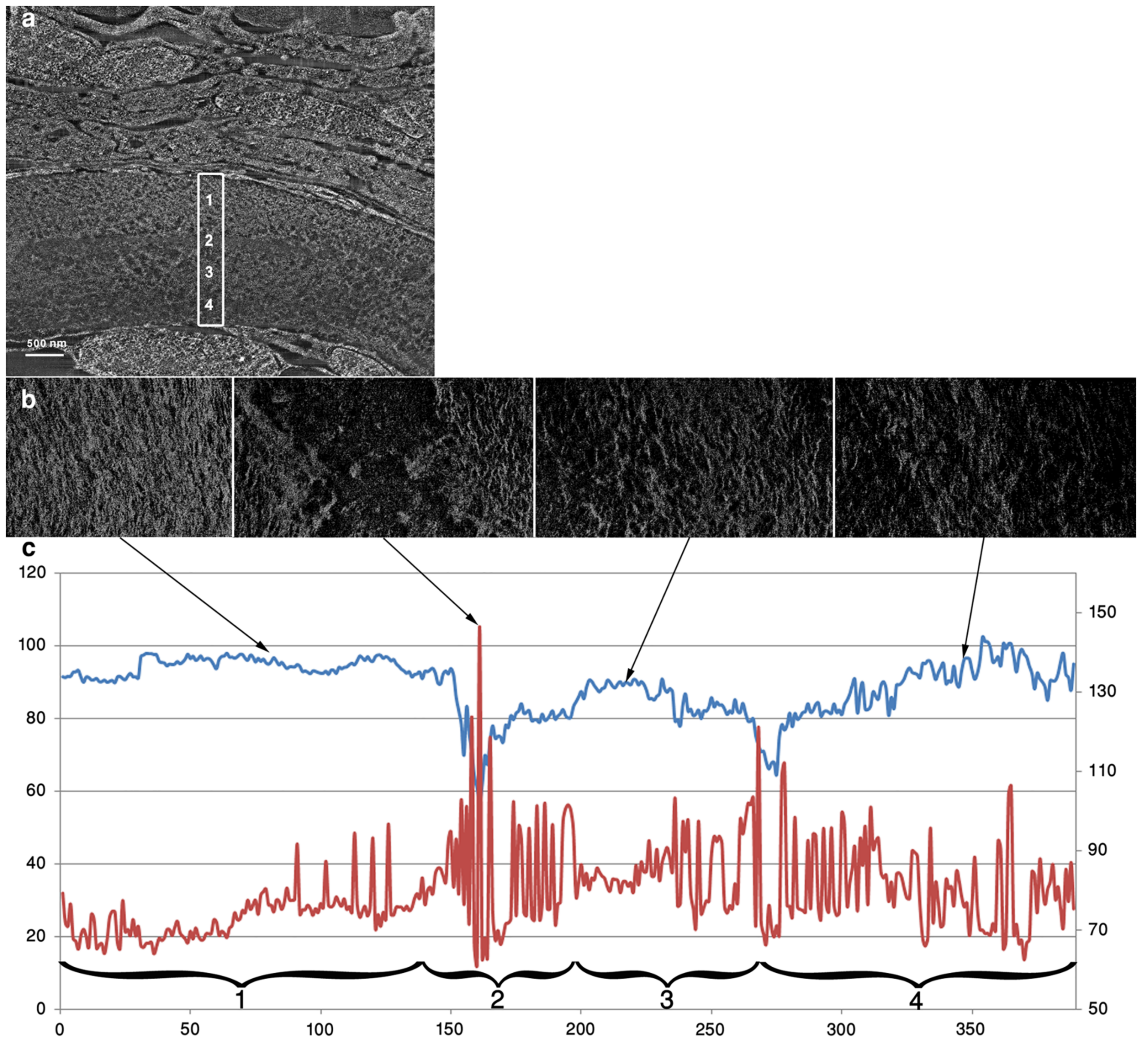
Directionality and dispersion analyses of the collagen fibrils in the tail bone at 30 dpf. **a**. FIB slice of the demineralized bone, observed in transverse plane. The rectangle spans the bone thickness. **b**. FIB slices characteristic of the different regions observed in the rectangle, observed in frontal plane, Slice 80 for region 1, slice 161 for region 2, slice 216 for region 3 and slice 346 for region 4. **c**. Direction (in blue) and dispersion (in red) plots of the collagen fibrils for each slice in the stack. The left vertical axis shows the azimuthal direction angle whereas the right shows the angular dispersion.

We also used FIB-SEM to characterize the collagen fibrils of the wild type demineralized lepidotrichia in terms of preferred orientation (direction) and the extent of preferred orientation (dispersion) [26,27] (Fig 5) (S3 Video). In the upper part of a more developped lepidotrichia, close to the outer surface (Fig 5a), the directionality values plotted against the slice number (Fig 5c) highlight 4 different zones. Zone 1, which is the most dorsal part, has the lowest dispersion (73.34° ± 5.73°) and the highest direction values (94.40° ± 2.32°), characteristic of an anistropic or aligned structure. Zone 2 has the highest dispersion (84.17° ± 16.48°) with the weakest direction values (80.05° ± 8.21°), implying a disordered structure. Zone 3 is more ordered, with the highest variation in the direction of the fibrils (84.29° ± 9.56°). Finally zone 4, which is the most ventral region, has a structure closer to zone 1, in terms of dispersion (80.49° ± 10.23°) and direction (89.42° ± 6.79°) values. The preferred orientation of the collagen fibers corresponds to the longitudinal axis of the lepidotrichia.

## Discussion

We show here that high resolution 3D imaging using confocal microscopy, quantitative FIB-SEM in the block surface serial imaging mode and quantitative high resolution laboratory based micro-CT of the zebrafish skeleton are powerful methods for revealing details of skeletal development, including the otoliths. High resolution micro-CT in particular, even with a laboratory micro-CT, could well prove to be a valuable tool for skeletal phenotype identification in both the imaging and quantitative modes.

In terms of identifying skeletal phenotypes, it is convenient to sub-divide skeletal formation into 3 processes: morphogenesis, bone collagen fibril architecture and bone mineralization. Mutations that affect morphogenesis should be expressed as a change in shape and volume of the bones. As micro-CT is capable of 3D imaging at relatively high resolution (0.6 μm) and can estimate bone volume, it is a powerful tool for detecting even small changes in the morphology of individual bones, including the very small bones of the zebrafish larvae. Micro-Ct has been used to study different bone parameters, including skeleton [22] and teeth [23,24]. Two different approaches are used: using a synchrotron based micro-CT or laboratory based micro-CT. The first method has a size of source of 800X30μm for a pixel size around 0.6 μm whereas the second has a size from 5 μm to 22 mm for a pixel size around 0.6 μm. The major difference between these two methods is linked to the exposure time, where the synchrotron based micro-CT need only 0.5 s for a sample whereas the laboratory based micro-CT requires 10 times more for the same one. But with time, this high resolution obtained using laboratory based micro-CT allows observations and characterizations of bone volume and densities from at least 10 dpf. Bone has a highly complex hierarchical structure [28]. Mammalian and fish bone has been shown to comprise two different materials: an ordered and a disordered material [26,27,29,30]. Mutations can affect many different aspects of the structure. Even high resolution micro-CT does not have the resolution to capture subtle changes in bone structure. We therefore show that the FIB SEM method can reveal detailed aspects of the structure, including the presence of ordered and disordered materials. Bone mineralization is a complex process that involves the uptake of ions from the environment, their transport to the bone, as well as remodeling of bone which releases ions back into circulation. These biomineralization pathways occur both intracellularly and in the extracellular environment, and involve the formation of solid mineral precursor phases [7]. An advantage of the micro-CT is that the amount of mineral in each bone can be mapped in 3D and quantified, and thus mutations that partially affect the biomineralization pathways might be identifiable.

Histological stains such as calcein and alizarin red for calcium and calcium containing mineral, alcian blue for glycosaminoglycans in cartilage, and von Kossa for divalent ions, are all useful for monitoring skeletal development [10,41]. Even if the calcein fluorescence signal is similar to the GFP signal, in this study we focused our analysis on the use of the calcein instead of the alizarin red to compare the results with those reported in the literature [10]. Unfortunately, after 30 dpf, the visualization of the skeleton *in vivo* is almost impossible using optical microscopy due to the presence of pigments and scales. Furthermore, we show here that in some cases vertebrae can be seen with calcein stain, but are absent when imaged by micro-CT. The calcein could well have stained precursor mineral bodies in the adjacent cells, as the mineralization of the vertebrae begins in the chordoblasts of the notochord [35]. Alternatively, it might be mapping high concentrations of free or bound ionic calcium in and between the cells close to the forming bone. In this respect the two methods are complementary.

Note too that the otoliths and the teeth are not generally stained *in vivo*, but calcein can be used to monitor growth increments [42]. The teeth of sacrificed zebrafish have been observed by alizarin red staining [43] or by using synchrotron micro-CT [22,23].

Radiography does image the mineralized bones and is certainly useful for identifying phenotypes that affect bone morphology [19,20]. However radiographic images are 2D projections of a 3D structure, and therefore subtle morphological changes in bone morphology and mineral density may not be identifiable.

Mutations that affect the otoliths may be readily discernible due to a different swimming behavior, as one function of otoliths is gravity perception [44,45]. It is thus of much interest to characterize the shapes and mineralization properties of the otoliths. Calcein does not reveal the otoliths, but they can be seen in detail using high resolution micro-CT.

The nacre mutant zebrafish is widely used for diverse studies [46] but also in another line, to have another line,the double recessive mutant with Roy (-/-), such as cancer [47,48], development [4,14,49–51] and behavior [52]. In all these studies the mutations responsible for pigmentation defects are not considered to disturb the wild type phenotype of the zebrafish, although this has been questioned [36]. In our study, we observed that *nacre* mutants display a delay in skeleton formation even during the first 7 days when environmental growth conditions are not generally thought to affect development (not shown here). We also noted using high resolution 3D imaging that *nacre* mutants do not possess mineral in their axial skeletons at 17 dpf, even though calcein clearly reveals what appear to be vertebrae. We do however note that at 30 dpf using both fluorescence and micro-CT imaging, *nacre* and wild fish have similar, but even then not identical skeletons. Four bones in the *nacre* mutant are different in terms of mineral density at 30 dpf. Significantly, 3 of these bones originate from the neural crest (ceratohyal bone, quadrate, pterosphenoid) and one from the mesoderm (parasphenoid) [53]. The comparison between the *nacre* mutant and the wild type is a good illustration of the potential of these techniques to identify phenotypes.

We used FIB-SEM to better characterize the 3D structure of the tail fin lepidotrichia bones. Even though the maximal resolution is higher in TEM compared to the FIB-SEM, this latter method allows us to locate the exact region of interest in 3 dimensions in relatively large volumes (tens of microns cubed). Furthermore the FIB SEM can be used in a correlative mode with other microscopes *(e*.*g*. fluorescence microscope) [54]. The FIB-SEM is thus a powerful method to characterize bone structure. In our study, the FIB-SEM allows us to identify layers in which the collagen fibrils are aligned in one direction, and layers in which there was no preferred alignment. Atkins et al also used FIB-SEM to study the structure of mature opercula in tilapia, and found the presence of both ordered and disordered bone material types [29]. In the operculum the aligned collagen fibrils continuously changed direction in a helical manner, and the thin disordered layers were interspersed inside the ordered structure. Mammalian bone also contains ordered and disordered bone materials [26,27,30]. These observations support the view that even at the bone structural level fish bone has the same basic structural motifs as mammalian bone.

In 1904, Goodrich used the term of lepidotrichia for both Actinopterygii and Sarcopterygii groups [55]. In the latter group, he observed that the lungfish have a specific elongated bone with a different structure called camptotrichia. These dermal bones are mineralized only in their superficial part and are unmineralized elsewhere with a disordered collagen [56]. These camptotrichia were first described as being potentially homologous to lepidotrichia [57], but later Geraudie and Meunier (1984), considered only the mineralized part to be equivalent to the lepidotrichia. Using our high resolution 3D imaging on the demineralized zebrafish tail fin, we observed an ordered structure similar to the camptotrichia: a mineralized outer part and a disordered structure on the inner part. These results confirm the homology between the lepidotrichia and the camptotrichia.

In the studies using transparent lines, even if the question was previously asked to know if the lack of pigments affects the biological processes [36], the transparency was not considered to disturb the wildtype phenotype of the zebrafish. In our study, we have shown that the nacre line, display a delay in growth and in the skeleton formation in term of date (S2 Fig). Indeed, when observed by high resolution 3D imaging, the zebrafish at 17 dpf did not present a mineral form in their axial skeleton. Using the comparison obtained with the fluorescence observations, and due to the variability coming from the delay in the growth, we conclude it is important to compare the specimens after 30 dpf, or to observe them in term of length of the body or the notochord. Nevertheless, even at this stage, where the total length is similar between the different types, the micro-CT revealed some differences in the biological processes. Indeed, at 30 dpf, the mineral density of some bones (the quadrate, the parasphenoid, the ceratohyal) found in the proximal part of the cranium or the otoliths (asteriscus, lapillus and sagitta) are much lower than the wild type. These bones could be interesting in the research of markers of abnormalities of skeleton development. Therefore, the albino type is not a “wild type” type. In the future, it would be interesting to observe if other organogenesis are affected.

In the *nacre* fish, we observed the presence of large mineralized aggregates in the nucleus pulposus cells of the notochord. The mineralization of the vertebrae always begins within the notochord sheet, in the chordoblasts, and then the perichordal bone is deposited [35]. In *nacre* mutants, these mineralized structures were found prior to the formation of the first axial skeletal element and in the nucleus pulposus. We do not exclude the possibility that these aggregates can be pathological, but they may also be part of the normal axial skeleton formation process, such as playing a role in the storage of the mineral until the beginning of the formation of the axial skeleton. It is conceivable that this stage could not be observed in the wild type because of the fast development of the axial skeleton, beginning around 4–5 dpf.

The presence of possibly analogous dense materials to those found in the zebrafish notochord was also observed in the sturgeon [58]. These structures were found in the core of the notochord, in the funiculus, but not within the notochord sheet. They are tubular in appearance and irregularly mineralized. Nevertheless, the authors also cannot exclude the possibility that these structures are pathological due to the breeding conditions in the sturgeon farms which differ from the wild. It would be interesting to determine if these structures are present in other fish such as the salmon that has a slow skeleton development and where the notochord is large [59].

In the zebrafish, three different chromatophores are defined: melanophores, iridiphores and xanthophores [60,61]. More than 100 genes are known to affect pigmentations when mutated [62,63]. Among these different mutant lines, various phenotypes are observed, such as ear development defects [63,64], and reduced overall size at 5 dpf [64,65]. Whitfield et al. showed that some mutations on genes affecting the otoliths can induce defects in other parts of the zebrafish, such as the chromatophores or the cartilaginous element of the jaw. The inner ear and cranial bones have a cranial neural crest origin, whereas chromatophores originate from the trunk neural crest cells [64,66,67]. In *nacre* mutants, we observed developmental abnormalities in the otoliths and in some cartilaginous bones of the jaw (ceratohyal bone, quadrate, pterosphenoid), in some dermal bones of the skull (parasphenoid and), and in the axial skeleton in the form of a delay in the formation of dermal bones (vertebrae) of mesodermal origin. The neural crest-mesoderm interface is described as a barrier that prevents the contact between the connective tissue precursors, but the mesoderm environment is involved in the regulation of cell movement [68]. *In vitro* experiments have shown interaction between the neural crest cells and the mesoderm for inner ear formation [69]. Our observations are consistent with the notion that the cranial neural crest, the trunk neural crest and the mesoderm strongly interact during skeleton development.

## Conclusions

The skeleton development is a complex system which requires a detailed approach using different high resolution 3D methods to define phenotypes. We show how the high resolution 3D imaging micro-CT and the FIB-SEM, do provide new insights into various aspects of zebrafish larval development, and may prove to be valuable tools for identifying phenotypes, both qualitatively and quantitatively.

## Supporting information

S1 Fig Zebrafish anatomy. **Side views of calcified labelled skull skeletal structures in developing wild type zebrafish larvae at 3, 5, 7, 10 and 14 dpf (D3,…) following calcein staining observed in fluorescence microscope**. Bop: Basioccipital process; Br1: Branchiostegal ray 1; Br2: Branchiostegal ray 2; Br3: Branchiostegal ray 3; Cb5: Ceratobranchial 5; Chb: Ceratohyal bone; Cl: Cleithrum; Dt: Dentary; Ep: Entopterygoid; Ex: Exoccipital; Hyo: Hyomandibula; La: Lapillus; Mp: Metapterygoid; Mx: Maxilla; Op: Opercle; Pt: Pterosphenoid; Qu: Quadrate; Ra: Retroarticullar; Sa: Sagitta; Sop: Subopercle; Vt: Vertebra.
(DOCX)

S2 Fig Development of the wild type skeleton based. **Side views of calcified skeletal structures in developing wild type (A, B) and nacre zebrafish larvae at 3, 5, 7, 10, 14 and 17 dpf (D3,…) and at 21, 24, 26, 28 and 30 dpf (D21,…) following calcein staining observed in fluorescence microscope (A, C), light microscope (B)**. Arrow: appearance of the first vertebra; *: zebrafish with a complete skeleton.
(DOCX)

S3 Fig Number of vertebrae in the wild type as a function of the notochord length. (DOCX)

S1 Video Micro-CT view of the bones in the 30 dpf wild type zebrafish showing only the head and first vertebrae of the axial skeleton. Pause 1: Note the 5 vertebrae bearing ribs, and between the vertebrae and the head, the Weberian apparatus (white arrow).
Pause 2: View from the dorsal surface of the head. The smooth and rounded large otoliths are the lapilli, the lentil-shaped bodies are the asterisci, and the sagittae cannot be seen.
The movie then zooms in to show the elongated teeth attached to the jaws (white arrows).
(MP4)

S2 Video FIB SEM in the SSV mode showing first the aligned stack of X images of a pair of hemi-cylindrical tail fin bones (lepidotrichia) in transverse section. The smooth high contrast objects are the dense mineralized bones. The pigment cells also produce high contrast due to the presence of heavy ions and stain. The pigment cells are enclosed inside a sack. After moving through the image stack, the dense contrasting materials are surface rendered to show their 3D structures.
Pause 1: the hemi-cylindrical shape of the bones can be recognized, and the pigment sack is seen to be highly elongated. Of particular interest is the”grey” elongated granular structure just below the upper left hemi-cylinder (white arrow).
Pause 2: After 90° rotation, this grey elongated granular structure is seen to be located below the entire upper hemi-cylinder (white arrow).
Next is a view through the stack of images starting from the dorsal surface with a pause showing the elongated granular structure located between two smooth dark collagen bundles (actinotrichia) (white arrow).
(MP4)

S3 Video FIB SEM in the SSV mode of a demineralized fin tail bone (lepidotrichia) showing the stack of X images in the transverse section. Pause 1: a cell and associated tissue (grey) overlain by the collagen fibrils isosurface rendered in yellow.
Collagen layer is rotated 90°.
Pause 2: transverse view through the demineralized bone section showing that the upper and lower surfaces are composed of aligned fibrils, whereas the center is dominated by disordered fibrils.
This is followed by a series of images from the stack starting with the dorsal surface and moving to the ventral surface. Note that the line in the center is an artifact.
Pause 3: end of the ordered zone.
Pause 4: end of the disordered zone.
Last sequence shows the ventral ordered layer.
(MP4)

S4 Video Micro-CT view of the head and upper axial skeleton of the nacre albino line at 30 dpf. By comparing to video 1 of the 30 dpf wild type it can be seen that micro-CT reveals all the bones, as well as the otoliths of the head and the vertebra. Many of these bones cannot be seen when stained by calcein. Note that the small dense fragments are from the ingested food.
(MP4)
