# Supplementary material for: Zebrafish skeleton development: High resolution micro-CT and FIB-SEM block surface serial imaging for phenotype identification
Source: PLoS One. 2017 Dec 8;12(12):e0177731. doi: 10.1371/journal.pone.0177731 (PMC5722281; doi:10.1371/journal.pone.0177731)

**Supplementary 1**

***Zebrafish anatomy***

The skeletal system of zebrafish is similar to the tetrapod system with a complex ossified skeleton that contains cartilage, bone and teeth, but no spongy bone and bone marrow [3]. In zebrafish, the skeleton can be divided into three parts: the cranium, the axial skeleton with its associated fins, and the fin tail [8]. The skull of the adult zebrafish is composed of 73 cranial bones, including 29 dermal bones, one membrane bone and 43 cartilage bones (Cubbage and Mabee, 1996; Eames et al., 2013). The first bones that begin to form during development are in the cranium. These bones form after 5 days post fertilization (dpf) [10,12] (**S1 Fig.**). In the cranium, the zebrafish has three pairs of otoliths: the sagitta, lapillus and asteriscus (**Video 1**). The lapillus was described as essential for the survival of the fish [30]. The sagitta and lapillus are first formed around 19 -22 hours post fertilization (hpf) [31], whereas the asteriscus is formed later (11-12 dpf) [32]. The three pairs of otoliths differ in their calcium carbonate mineral polymorphs: while the sagitta and lapillus are composed of aragonite, the asteriscus is composed of vaterite [33]. The aragonite and vaterite minerals play a role in the perception of sound and balance [34].

The axial skeleton is separated into 4 regions: (1) the Weberian or cervical vertebrae, including the first 4 vertebrae and the Weberian apparatus (**Video 1**); (2) the precaudal vertebrae, formed by 10 vertebrae bearing ribs; (3) the caudal vertebrae, made up of 14 vertebrae with hemal arches; (4) and the caudal fin vertebrae, formed by 3 vertebrae [8,35]. The bones in the skeleton have both dermal and cartilagenous origins, in addition to a perichordal origin for the centra [35]. At 7 dpf the trunk region of the axial skeleton is observed (based on Alizarin Red staining), with the initial formation of vertebrae 3 and 4 centra [8,35]. Then the first two vertebrae are mineralized at the same time as vertebra 5. The remaining vertebrae mineralize after this. These centra are mineralized first in a ‘ring’ shape which then expands in the antero-posterior axis [35]. The fin tail is formed by 3 caudal vertebrae, 11 other bones and 19 fin rays [8]. The formation of the tail begins with the ural 1 at 17 dpf and is fully formed at 30 dpf [10].

.


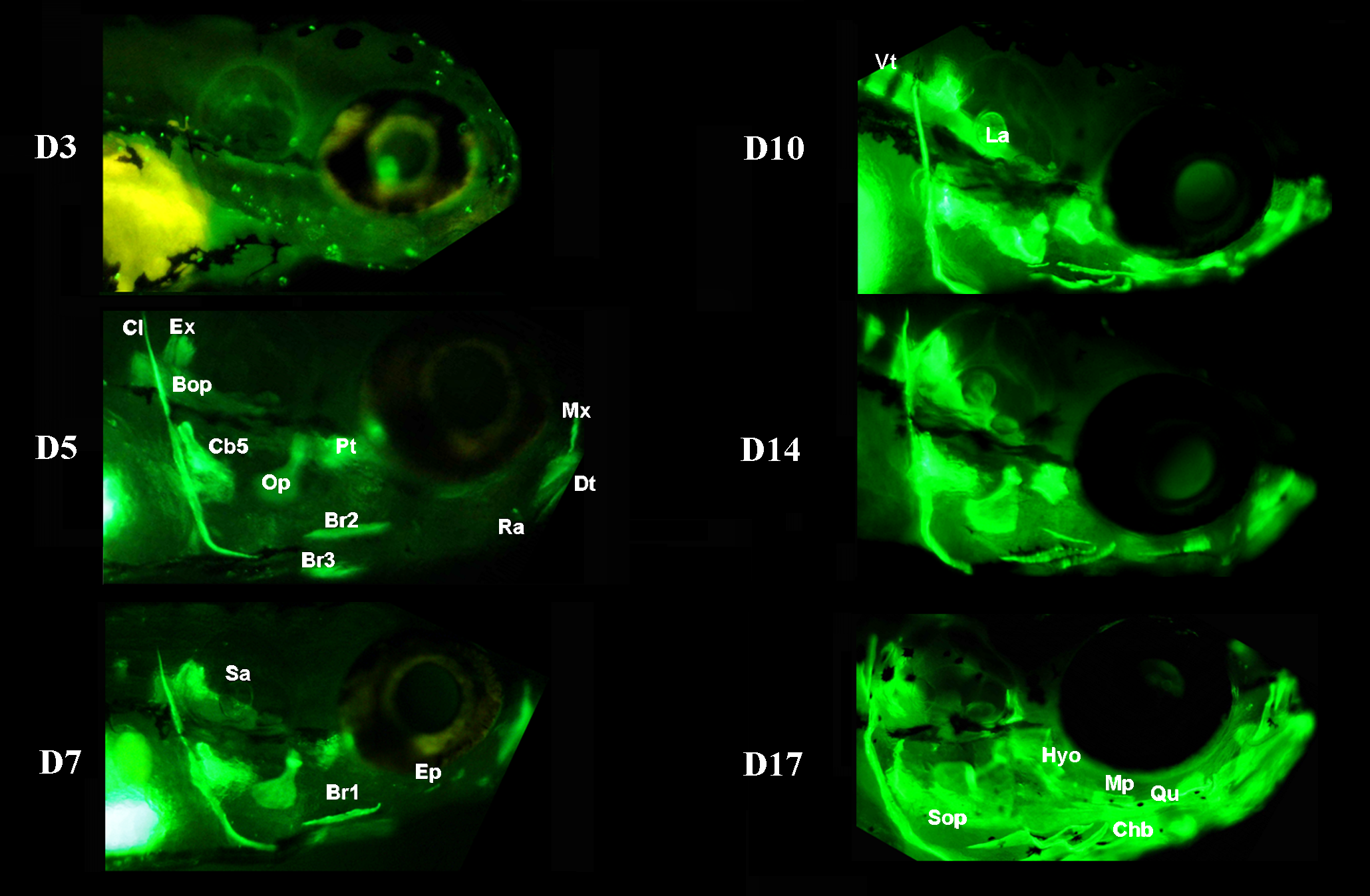

Supplement: S1 Fig — Side views of calcified labelled skull skeletal structures in developing wild type zebrafish larvae at 3, 5, 7, 10 and 14 dpf (D3,…) following calcein staining observed in fluorescence microscope. Bop: Basioccipital process; Br1: Branchiostegal ray 1; Br2: Branchiostegal ray 2; Br3: Branchiostegal ray 3; Cb5: Ceratobranchial 5; Chb: Ceratohyal bone; Cl: Cleithrum; Dt: Dentary; Ep: Entopterygoid; Ex: Exoccipital; Hyo: Hyomandibula; La: Lapillus; Mp: Metapterygoid; Mx: Maxilla; Op: Opercle; Pt: Pterosphenoid; Qu: Quadrate; Ra: Retroarticullar; Sa: Sagitta; Sop: Subopercle; Vt: Vertebra. (DOCX) [file pone.0177731.s001.docx]
