# Supplementary material for: Zebrafish skeleton development: High resolution micro-CT and FIB-SEM block surface serial imaging for phenotype identification
Source: PLoS One. 2017 Dec 8;12(12):e0177731. doi: 10.1371/journal.pone.0177731 (PMC5722281; doi:10.1371/journal.pone.0177731)

**Supplementary 2**

***Development of the skeleton based on calcein fluorescence microscopy***

Living wild type and nacre type zebrafish were immersed in a calcein solution and observed under a fluorescence microscope from 3 dpf to 30 dpf in order to monitor skeletal development. The 3dpf nacre mutant cranium does fluoresce strongly, as opposed to the wild type cranium. Examination at high magnification reveals that the nacre cranial bones are not fluorescent, and that for some unknown reason the fluorescence is diffused over the entire cranium (**S2 Fig.)** At 5 dpf, a fluorescence signal is observed in the cranium of both wild type and albino zebrafish. Some cranial elements are clearly observable, such as the cleithrum, the basioccipital process, the exoccipital, the ceratobranchial 5, the opercle, the pterosphenoid, the branchiostegal ray 2 and 3, the retroarticular, the maxilla and the dentary. The axial skeleton is observed only in the wild type at 5dpf, but not in either of the albino, as shown by the staining in the third vertebra, and a fainter staining in the second, fourth and fifth vertebrae. (**S2 Fig. arrow**).

The wild type axial skeleton including the tail fin bones is fully developed by 17dpf, whereas in the axial skeletons of the mutant only vertebrae 3 to 9 for the nacre type are observed. At 17 dpf, in the wild type, all the cranium bones and the complete axial skeleton including the tail fin bones are observed. (**S2 Fig.)**. In addition, the first ribs and some elements of the Weberian apparatus are also observed. In the albino fish, the entire cranium dermal bones are observed only at 24 dpf, and the complete axial skeleton at 26 dpf. This delayed skeletal development in the albinos revealed by calcein fluorescence appears to be a major difference relative to the wild type. At 30 dpf all the cranium bones and the complete axial skeletons are observed both in the wild type and in the albino. At this stage, the ribs, the dorsal and anal fins and elements belonging to the Weberian apparatus are also labeled by calcein fluorescence.

Remarkably, at 30 dpf, with this *in vivo* calcein staining, it is difficult to observe the otoliths or the teeth in the ceratobranchial 5.

We then quantified the development of the skeleton as revealed by calcein using meristic traits. We measured the distance from the cleithrum to the posterior tip of the notochord (until 17 dpf), or after the bones are formed, to the caudal peduncle and referred to this as notochord length. We compared the number of vertebrae revealed by calcein as a function of notochord length (**S3 Fig.**). Using this approach we could divide development into three stages: when no vertebrae are formed and the notochord length is less than ± 3 mm, when vertebrae develop (notochord length 3 to 4 mm) and when the entire axial skeleton is visible (notochord length greater than 4 mm).


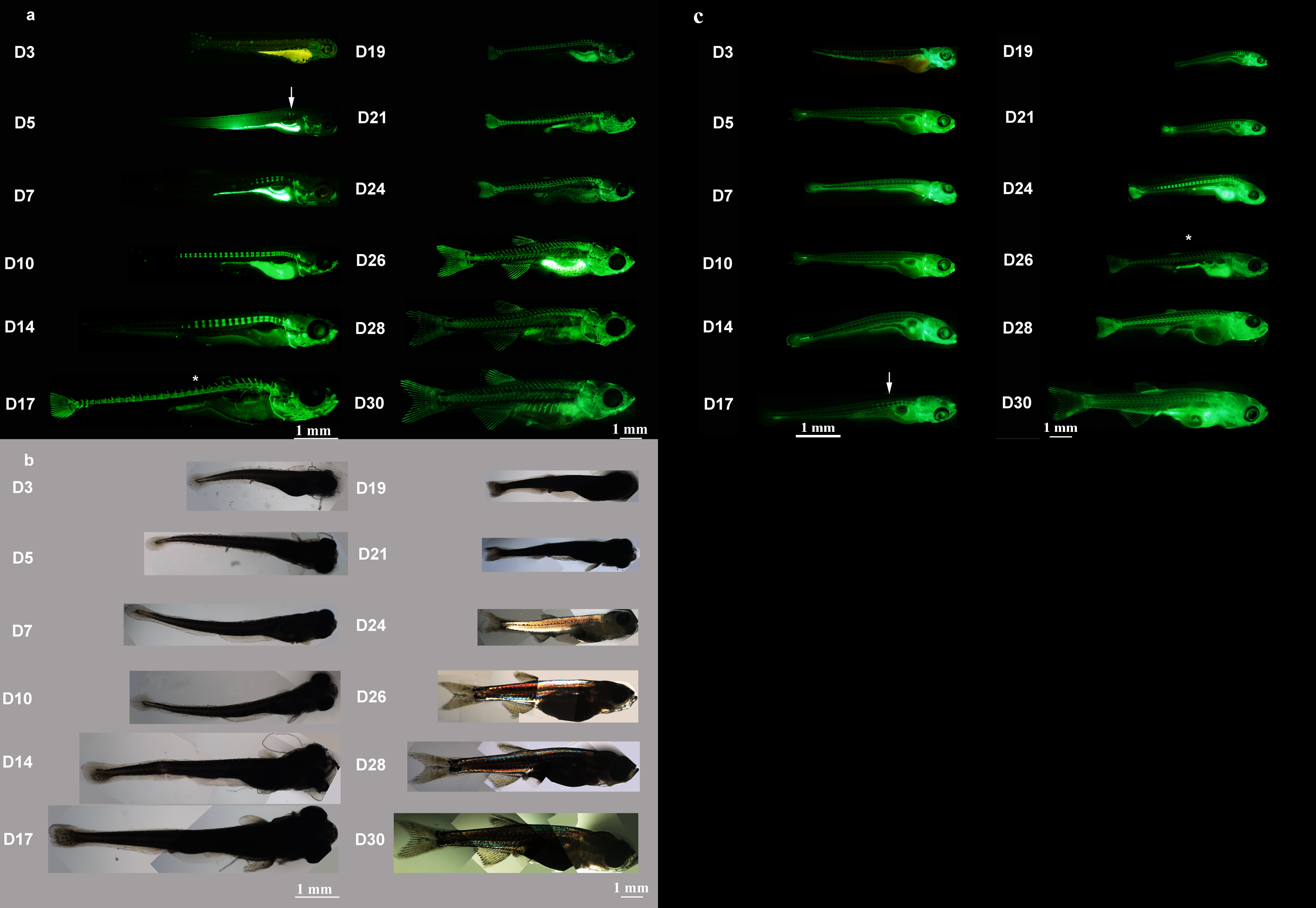

Supplement: S2 Fig — Side views of calcified skeletal structures in developing wild type (A, B) and nacre zebrafish larvae at 3, 5, 7, 10, 14 and 17 dpf (D3,…) and at 21, 24, 26, 28 and 30 dpf (D21,…) following calcein staining observed in fluorescence microscope (A, C), light microscope (B). Arrow: appearance of the first vertebra; *: zebrafish with a complete skeleton. (DOCX) [file pone.0177731.s002.docx]
