# Supplementary material for: Zebrafish skeleton development: High resolution micro-CT and FIB-SEM block surface serial imaging for phenotype identification
Source: PLoS One. 2017 Dec 8;12(12):e0177731. doi: 10.1371/journal.pone.0177731 (PMC5722281; doi:10.1371/journal.pone.0177731)
Supplement: S3 Fig — (DOCX) [file pone.0177731.s003.docx]

**Supplementary 3: Number of vertebrae in the wild type as a function of the notochord length.**
